# Patient-derived avian influenza A (H5N6) virus is highly pathogenic in mice but can be effectively treated by anti-influenza polyclonal antibodies

**DOI:** 10.1038/s41426-018-0113-2

**Published:** 2018-06-13

**Authors:** Weiqi Pan, Haojun Xie, Xiaobo Li, Wenda Guan, Peihai Chen, Beiwu Zhang, Mincong Zhang, Ji Dong, Qian Wang, Zhixia Li, Shufen Li, Zifeng Yang, Chufang Li, Nanshan Zhong, Jicheng Huang, Ling Chen

**Affiliations:** 1 State Key Laboratory of Respiratory Disease, The First Affiliated Hospital of Guangzhou Medical University, Guangzhou, China; 2 Health Quarantine Laboratory, Guangdong Inspection and Quarantine Technology Center, Guangzhou, China; 3 Guangzhou Institutes of Biomedicine and Health, Chinese Academy of Sciences, Guangzhou, China

## Abstract

Highly pathogenic avian influenza A (H5N6) virus has been circulating in poultry since 2013 and causes sporadic infections and fatalities in humans. Due to the re-occurrence and continuous evolution of this virus subtype, there is an urgent need to better understand the pathogenicity of the H5N6 virus and to identify effective preventative and therapeutic strategies. We established a mouse model to evaluate the virulence of H5N6 A/Guangzhou/39715/2014 (H5N6/GZ14), which was isolated from an infected patient. BALB/c mice were inoculated intranasally with H5N6/GZ14 and monitored for morbidity, mortality, cytokine production, lung injury, viral replication, and viral dissemination to other organs. H5N6/GZ14 is highly pathogenic and can kill 50% of mice at a very low infectious dose of 5 plaque-forming units (pfu). Infection with H5N6/GZ14 showed rapid disease progression, viral replication to high titers in the lung, a strongly induced pro-inflammatory cytokine response, and severe lung injury. Moreover, infectious H5N6/GZ14 could be detected in the heart and brain of the infected mice. We also demonstrated that anti-influenza polyclonal antibodies generated by immunizing rhesus macaques could protect mice from lethal infection. Our results provide insights into the pathogenicity of the H5N6 human isolate.

## Introduction

Since its first isolation in Laos in 2013, H5N6 has been subsequently reported in Vietnam and China and more recently in Korea^1–4^. Approximately 155 outbreaks at poultry farms have been reported in 18 provinces across China with sporadic human infections, including 16 cases of human infections with a high fatality rate (9/16 cases, 56.3%) since 2014, a fatality rate that is higher than that reported for H5N1 (52.8%) and H7N9 (39.1%)^5,6^. The continued and overwhelming circulation of the H5N6 virus instead of the H5N1 virus in poultry in southern China poses a considerable threat to public health and increases the risks for human infections worldwide through international travel and migratory wild birds^7^. Therefore, it is necessary to better understand the pathogenicity of the H5N6 human isolate in mammals and identify prevention and therapeutic strategies.

In this study, we investigated the pathogenicity of the human-derived H5N6 isolate A/Guangzhou/39715/2014 (H5N6/GZ14) in BALB/c mice. This virus was isolated from a 59-year-old male patient with a history of visiting poultry markets who survived severe acute respiratory distress syndrome (ARDS) in our hospital (the First Affiliated Hospital of Guangzhou Medical University, Guangzhou, China)^8^. We evaluated viral replication in the lung, viral dissemination in the extrapulmonary organs, pulmonary histopathology, and the level of cytokines and chemokines in the lungs of infected mice. By immunizing rhesus macaques with inactivated recombinant avian influenza A viruses, we generated an anti-influenza immunoglobulin G (IgG) consisting of polyclonal antibodies with neutralizing activity against H5N6/GZ14. We demonstrated that H5N6/GZ14-infected mice could be effectively protected by anti-influenza polyclonal antibodies.

## Results

### The patient-derived H5N6/GZ14 isolate was highly lethal in BALB/c mice

**Fig. 1:**
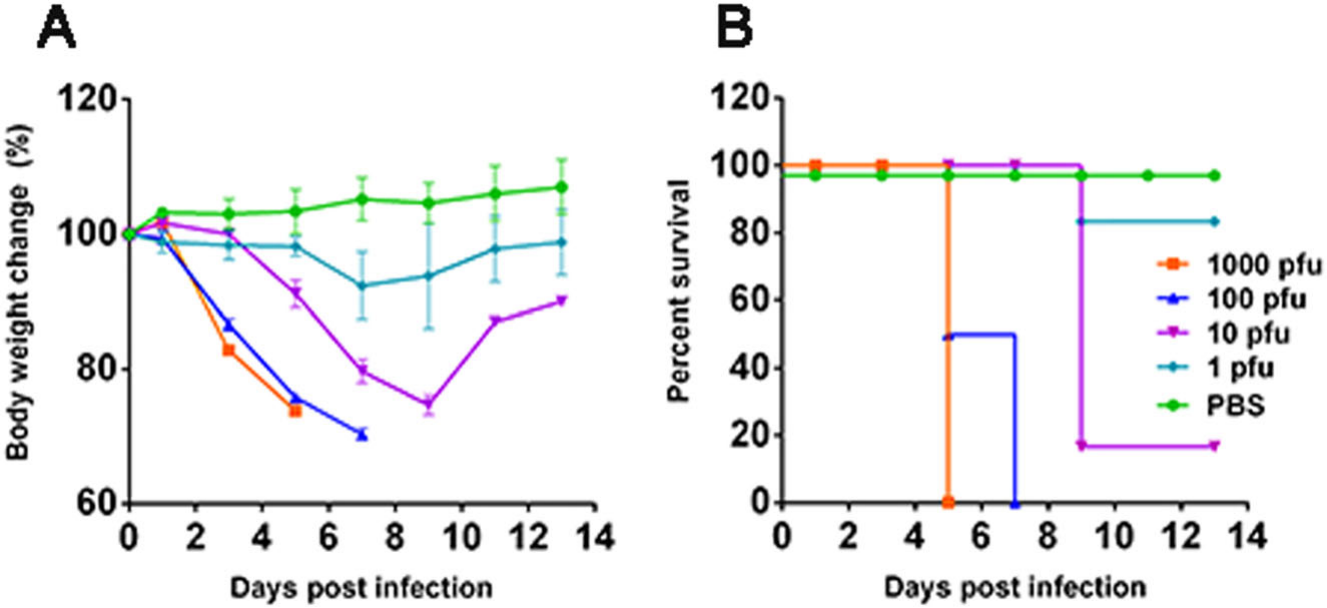
Infection of the patient-derived H5N6/GZ14 isolate in mice. Eight-week-old female BALB/c mice (*n* = 6) were anesthetized with isoflurane and infected intranasally with 1, 10, 100, or 1000 pfu of H5N6/GZ14. The mice were monitored for 14 days. **a** The body weights were measured every other day. The results from each group and each time point are expressed as the mean ± standard deviations (SD). **b** Survival of infected mice. Mice were euthanized when body weight loss exceeded 25% of the original weight

Because there was no prior information regarding the virulence of human-infected H5N6/GZ14 in mice, we attempted to infect 8-week-old female BALB/c mice intranasally with 1, 10, 100, and 1000 plaque-forming units (pfu) of H5N6/GZ14. The mice were monitored for signs of illness, weight loss, and mortality for up to 14 days. The mice were euthanized when the weight loss exceeded 25% of the original body weight. Mice infected with 1000 pfu started to show signs of illness, including a ruffled coat and decreased activity, at 2 days post infection (dpi). Mice had an average of 17% weight loss at 3 dpi, and none survived at 5 dpi (Fig. 1a, b). Mice infected with 100 pfu showed signs of illness at 3 dpi. Three out of six mice in this group were euthanized at 5 dpi due to weight loss >25%. The remaining three mice were euthanized at 7 dpi due to weight loss >25% (Fig. 1a, b). Mice infected with 10 pfu showed signs of illness and weight loss at 5 dpi, which was delayed 2~3 days compared to mice infected with 1000 and 100 pfu. Five mice in this group were euthanized at 9 dpi due to weight loss >25% (Fig. 1a, b). Mice infected with 1 pfu showed an average weight loss of 10% at 7 dpi, but most mice survived and regained their body weight, and only one mouse had to be euthanized at 9 dpi due to weight loss >25% (Fig. 1a, b). The calculated 50% mouse lethal dose (MLD_50_) of H5N6/GZ14 is 5 pfu. This result revealed that the patient-derived H5N6/GZ14 isolate, without prior adaptation in mice, has a very high degree of lethality in BALB/c mice.

### The patient-derived H5N6/GZ14 isolate replicated efficiently in mouse lung and can disseminate systemically

To evaluate the viral replication and dissemination of H5N6/GZ14 in mice, we infected mice with H5N6/GZ14 at 10 MLD_50_ (50 pfu). Three mice were sacrificed per day at 1, 3, and 5 dpi for evaluation of viral replication in vivo. H5N6/GZ14 appeared to replicate rapidly to a high titer (3.55 × 10^6^ pfu (10^6.55±0.88^ pfu)/g tissue) in the lungs at 1 dpi, reached a peak (4.90 × 10^7^ pfu/g (10^7.69±0.05^ pfu/g)) at 3 dpi, and remained at a high level at 5 dpi (Table 1). To further determine whether H5N6/GZ14 has any extrapulmonary dissemination following infection, we investigated the viral titers in the heart, liver, spleen, kidney, brain, and serum by a plaque formation assay. At 1 dpi, no virus could be detected in the organs other than the lung of infected mice. At 3 dpi, two out of three mice had detectable virus in the heart, with an average titer of 1.12 × 10^5^ pfu/g (10^5.05^ pfu/g). At 5 dpi, all of the mice had detectable virus in the heart, with an average titer of 2.88 × 10^4^ pfu/g (10^4.46±0.07^ pfu/g). At 3 and 5 dpi, one out of three mice had detectable virus in the brain, with a titer at 2.69 × 10^2^ pfu/g (10^2.43^ pfu/g) and 5.50 × 10^3^ pfu/g (10^3.74^ pfu/g), respectively (Table 1). No infectious viruses could be detected in the blood, liver, spleen, and kidney. We also examined the relative amounts of H5N6 virus genome in the serum samples by real-time quantitative PCR (qPCR). Analysis of the serum samples collected at 1, 3, and 5 dpi showed an average of 110 viral genome copies per 100 μl of serum, which indicated the presence of viremia in mice infected with H5N6/GZ14 at 10 MLD_50_ (50 pfu).

**Table 1:** Replication of influenza H5N6/GZ in mice

Days	Virus titer in tissue^a^ (mean log_10_ pfu/g ± SD)			Virus detected in organs^c^		
	Lung	Heart	Brain	Lung	Heart	Brain
1	6.55 ± 0.88	**—** ^b^	**—**	3/3^d^	0/3	0/3
3	7.69 ± 0.05	5.05	2.43	3/3	2/3	1/3
5	7.04 ± 0.05	4.46 ± 0.07	3.74	3/3	3/3	1/3

^a^Mice were infected with 10 MLD_50_ (50 pfu)

^b^— represents virus was not detected in samples

^c^There was no detectable virus in liver, spleen, and kidney

^d^Number of mice had detectable virus in tissues per three mice

### H5N6/GZ14 infection resulted in severe lung injury and elevation of pro-inflammatory cytokines and chemokines in the lungs of infected mice

**Fig. 2:**
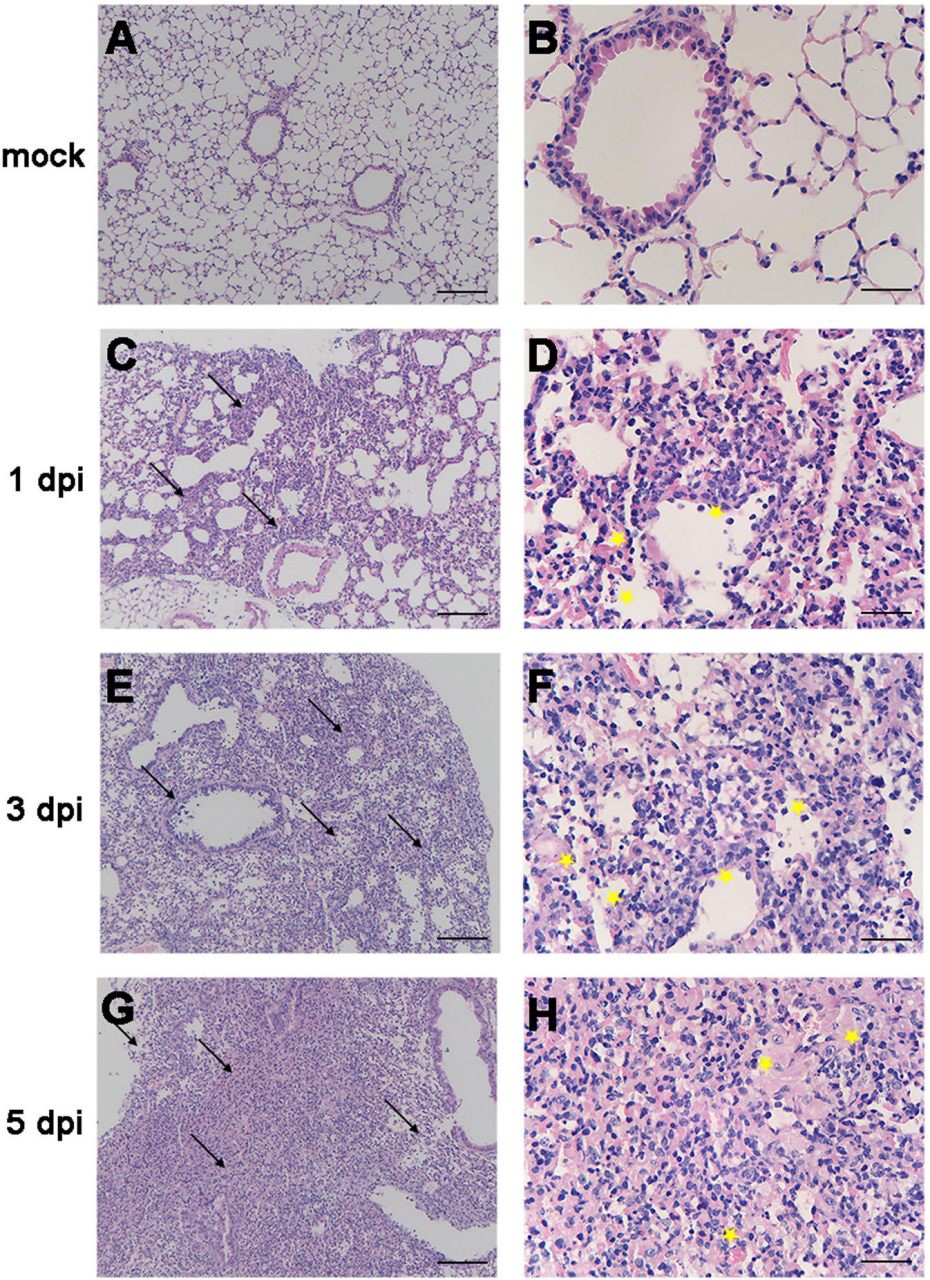
Histopathology of the lungs from H5N6/GZ14-infected mice. **a**, **b** Eight-week-old female BALB/c mice without infection. **c**–**h** Eight-week-old female BALB/c mice infected with 10 MLD_50_ (50 pfu) of H5N6/GZ14. Lung tissue sections were stained with hematoxylin-eosin and analyzed under a light microscope. At 1 dpi, inflammatory cells (arrows) could be observed around the bronchi (**c**, **d**). The airways showed small volumes of exudates with edema fluid mixed with erythrocytes and inflammatory cells (asterisks). **e**, **f** At 3f dpi, the lungs had high numbers of inflammatory cells (arrows), bronchial epithelial intracellular edema, and necrosis with necrotic epithelium sloughing into the airway spaces (asterisks). **g**, **h** At 5 dpi, severe pulmonary parenchyma consolidation was observed. Increased accumulation of inflammatory cells and necrotic tissue debris (arrows) was observed in the lung parenchyma. The alveoli were completely filled with edema and hemorrhages (asterisks). Scale bars = 100 μm (**a**, **c**, **e**, **g**) and 25 μm (**b**, **d**, **f**, **h**)

To determine the effect of H5N6/GZ14 infection on the lungs, we performed a histopathological examination of formalin-fixed, paraffin-embedded lung sections stained with hematoxylin and eosin. Histopathologic changes appeared as early as 1 dpi, with inflammatory cells noted around the observed bronchi. Some airways showed small volumes of exudates. The alveolar walls were thickened, and the alveolar lumens were flooded with edema fluid mixed with erythrocytes and inflammatory cells (Fig. 2c, d). At 3 dpi, H5N6/GZ14-infected mice had severe bronchial inflammation, bronchial epithelial intracellular edema, and necrosis with necrotic epithelium sloughing into the airway spaces (Fig. 2e, f). Severe pulmonary parenchyma consolidation was observed at 5 dpi. Increased accumulation of inflammatory cells, mainly neutrophils, macrophages, lymphocytes, and necrotic tissue debris, was observed in the lung parenchyma. Alveoli were completely filled with edema and hemorrhages (Fig. 2g, h). This result revealed that H5N6/GZ14 infection resulted in severe lung injury.

**Fig. 3:**
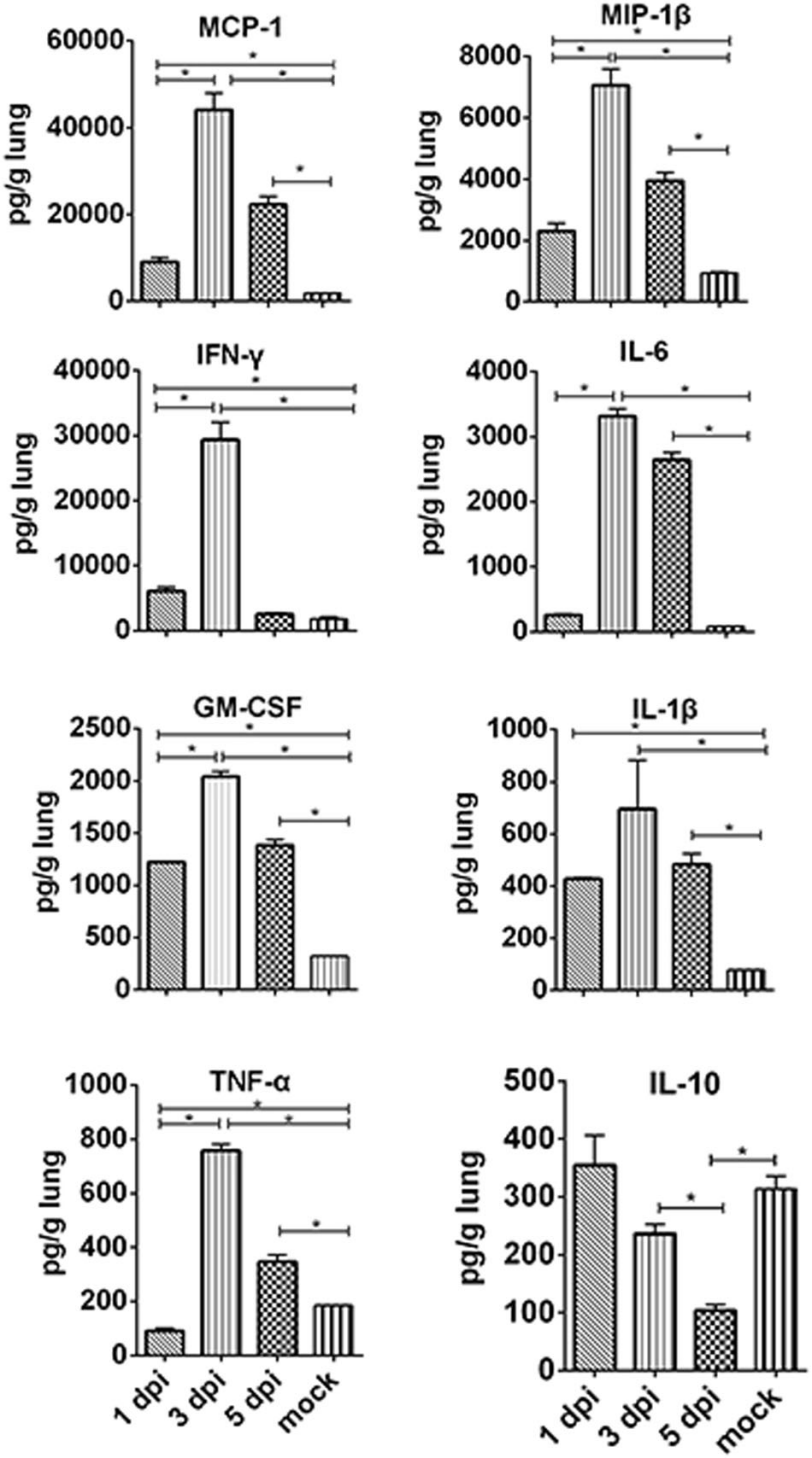
Pro-inflammatory cytokines and chemokines in the lungs of H5N6/GZ14-infected mice. The lung tissues were collected and homogenized from H5N6/GZ14-infected mice at 1, 3, and 5 dpi (*n* = 3). The levels of cytokines and chemokines in the lung homogenates (pg/g of lung tissue) were determined by ELISA. The results from each time point are expressed as the mean ± SD. *A significant difference (*P < 0.05)

To understand the relationship of disease progression and the level of pro-inflammatory cytokines and chemokines in the lungs, we collected lungs from mice infected with 10 MLD_50_ of H5N6/GZ14 at 1, 3, and 5 dpi. Enzyme-linked immunosorbent assay (ELISA) was used to measure seven pro-inflammatory cytokines and chemokines and one anti-inflammatory cytokine in the lung homogenates. At 1 dpi, the levels of the pro-inflammatory cytokines monocyte chemotactic protein 1 (MCP-1), macrophage inflammatory protein 1β (MIP-1β), gamma interferon (IFN-γ), granulocyte-macrophage colony-stimulating factor (GM-CSF), and interleukin 1β (IL-1β) significantly increased. These cytokines, interleukin 6 (IL-6) and tumor necrosis factor alpha (TNF-α), were elevated to the highest level at 3 dpi. At 5 dpi, MCP-1, MIP-1β, IL-6, GM-CSF, IL-1β, and TNF-α remained at a high level. In contrast, the level of the anti-inflammatory cytokine interleukin 10 (IL-10) decreased significantly at 3 and 5 dpi (Fig. 3). This result showed that H5N6/GZ14 infection could elevate pro-inflammatory cytokines and chemokines in the lungs.

### Mice infected with H5N6/GZ14 could be rescued by treatment with anti-influenza polyclonal antibodies generated from immunized rhesus macaques

**Fig. 4:**
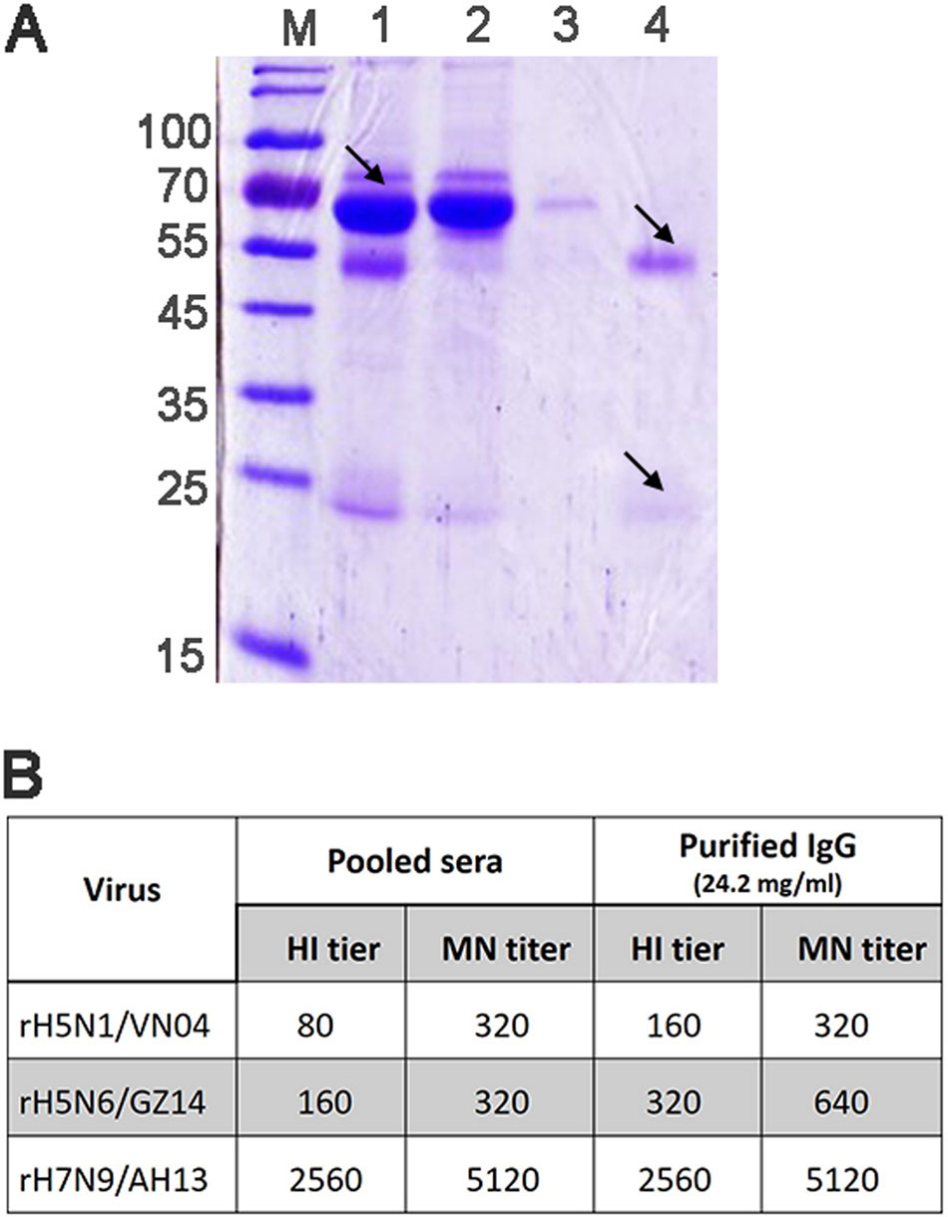
Coomassie Blue staining after SDS-PAGE and the antibody titer of immunized rhesus macaque sera and purified IgG. **a** Coomassie Blue staining after SDS-PAGE of macaque serum and purified IgG. M molecular weight markers (kDa). Lane 1: pooled hyperimmune rhesus macaque serum (diluted 500-fold). The filtered start serum mainly contains albumin (arrow). Lane 2: the flow through pool and unbound material (diluted 50-fold). Albumin and other proteins were removed and could be observed in the flow through pool. Lane 3: the effluent and column wash. Lane 4: the eluted and purified IgG (diluted 1000-fold). The IgG heavy chain (~50 kDa) and light chain (~25 kDa) (arrows) could be observed in the pooled elution buffer without albumin and other proteins. **b** HI and the MN neutralizing antibody titer of the pooled hyperimmune sera and the purified polyclonal IgG antibody. Three recombinant viruses, rH5N1/VN04, rH5N6/GZ14, and rH7N9/AH13, were used to immunize the rhesus macaques. HI titers are presented as the reciprocal value of the highest serum dilution that inhibited hemagglutination. MN titers are presented as the reciprocal value of the highest serum dilution that conferred 50% neutralization of 100 TCID_50_ of the virus

Previous studies have shown that treatment of severe H5N1 or H1N1 infections with convalescent plasma containing neutralizing antibodies to these influenza viruses could reduce the respiratory tract viral load, the serum cytokine response, and mortality^9,10^. However, there are no available human donors from H5N6 natural infection, and survivors are rare. Therefore, to obtain antibodies that could neutralize H5N6/GZ14, we generated an anti-influenza IgG that consists of polyclonal antibodies from immunized rhesus macaques. Rhesus macaques were immunized with three inactivated recombinant (6 + 2) viruses in the A/Puerto Rico/8/1934 (*PR8*) gene background, including rH5N1/VN04, rH7N9/AH13, and rH5N6/GZ14. Fifty milliliters of hyperimmune sera was collected and pooled for IgG purification. The pooled hyperimmune sera had hemagglutination inhibition (HI) and the neutralizing antibody against three recombinant viruses: rH5N6/GZ14; rH7N9/AH13; and rH5N1/VN04. Protein A-Sepharose affinity chromatography was used to purify the IgG and to remove serum albumin and other proteins (Fig. 4a). The antibody heavy chain (~50 kDa) and light chain (~25 kDa) of purified anti-influenza IgG could be observed without contamination from other proteins (Fig. 4a). After purification, a total volume of 27 ml was collected containing 24.3 mg of protein/ml, an HI antibody titer at 1:320, and a neutralizing antibody titer at 1:640 against recombinant rH5N6/GZ14 virus (Fig. 4b).

**Fig. 5:**
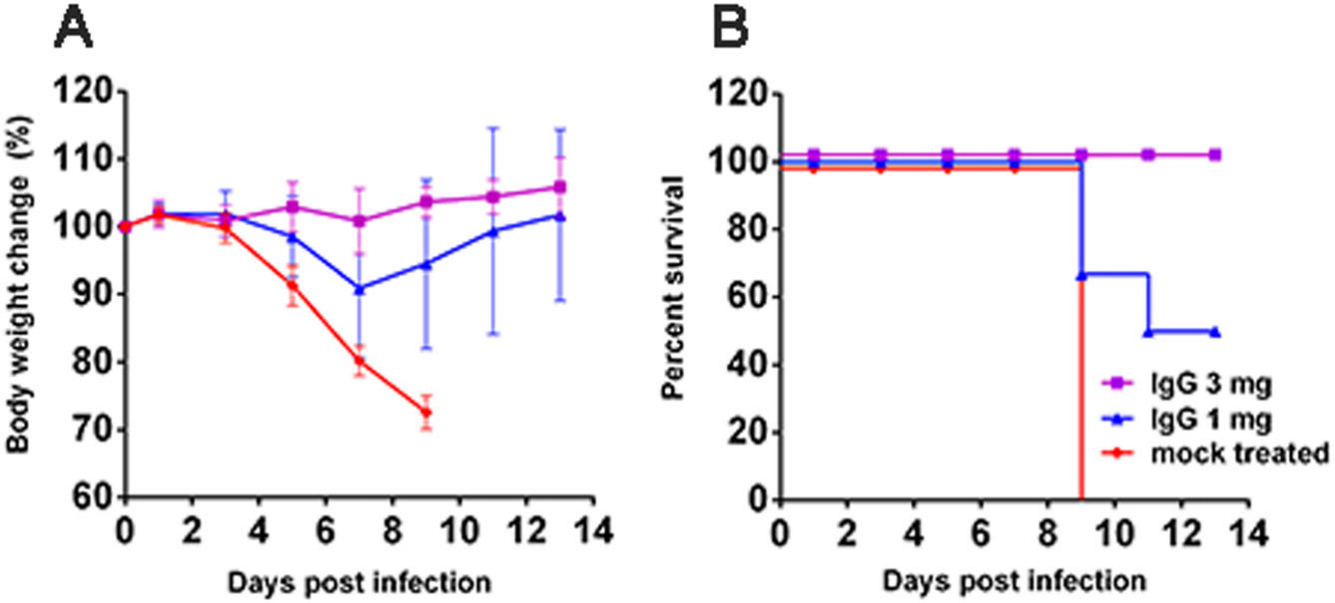
Efficacy of anti-influenza polyclonal antibodies for treating H5N6/GZ14-infected mice. Female 8-week-old BALB/c mice (*n* = 6) were intranasally infected with 2 MLD_50_ (10 pfu) of H5N6/GZ14. One day later, the mice were treated with an intraperitoneal injection of either 1 or 3 mg of anti-influenza IgG/mouse. The mock-treated mice received an intraperitoneal injection of 3 mg of purified unrelated IgG. **a** The body weights were measured every other day. The results from each group and each time point are expressed as the mean ± SD. **b** Survival of infected mice. Mice were euthanized when body weight loss exceeded 25% of the original weight

To evaluate whether the highly pathogenic H5N6/GZ14 infection in mice could be treated with anti-influenza polyclonal antibodies, we first infected BALB/c mice intranasally with 2 MLD_50_ of H5N6/GZ14. One day after infection, the mice were treated with purified IgG at either 1 or 3 mg/mouse via a single intraperitoneal injection. All of the mice receiving 3 mg of purified IgG survived lethal infection without obvious signs of illness. Mice receiving 1 mg of purified IgG also exhibited 50% survival with less weight loss. All of the mice receiving 3 mg of an unrelated IgG succumbed to infection by 9 dpi (Fig. 5). Therefore, lethal infection with H5N6/GZ14 in mice could be rescued using anti-influenza polyclonal antibodies generated by immunizing rhesus macaques with related influenza viruses.

## Discussion

In this study, we reported that H5N6/GZ14 isolated from a hospitalized patient who developed ARDS exhibited high virulence and severe disease phenotypes in BALB/c mice with an MLD_50_ value as low as 5 pfu. We also demonstrated that virulent disease progression was effectively inhibited using primate-derived anti-influenza polyclonal antibodies generated by immunizing rhesus macaques with related influenza viruses.

Over the past several years, highly pathogenic avian influenza H5N6 viruses have been circulating in poultry and have caused severe infections in humans at a high fatality rate comparable to that of H5N1 and H7N9 avian influenza A viruses^11,12^. BALB/c mice have been used to study the pathogenesis of and conduct antiviral therapeutic assessments for several influenza A viruses, including H5N1 and H7N9^13,14^. So far, there are no reports on the study of H5N6 human isolates in a mouse model. There are several studies in mice on the pathogenicity and transmissibility of the H5N6 virus isolated from birds^15–17^. In one study, the pathogenicity of three H5N6 avian isolates was evaluated, and the results varied from mildly pathogenic to highly pathogenic in mice^15^. Sequential passaging in mice using an H5N6 isolate from birds showed that E627K on PB2 played an important role in adaptation of the H5N6 virus to mice, which is similar to that reported for the H5N1, H9N2, and H6N1 influenza viruses^16–19^. In our study, the human-infected isolate, H5N6/GZ14, contains multiple basic amino acids at a hemagglutinin (HA) cleavage site and the mammalian adaptation mutation PB2-E627K, suggesting that this H5N6/GZ14 has high pathogenicity in mammals, which was also demonstrated in the infected patient^20^. Therefore, it would be interesting to test if the patient-derived H5N6 could infect mice, and if so, what the degree of virulence would be and whether the infection could be treated.

We found that the patient-derived isolate H5N6/GZ14, without prior adaptation in mice, is highly virulent in BALB/c mice. Disease progression and the course of infection were very rapid. Most importantly, H5N6/GZ14 spread beyond the respiratory tract to other organs, with infectious virus detected in the brain and heart at 5 dpi. Detection of the viral RNA in the serum samples from H5N6/GZ14-infected mice suggested that the dissemination of H5N6/GZ14 from the respiratory tract to other organs may be related to viremia that occurred early in infection.

In our study, the MLD_50_ of H5N6/GZ14 was only 5 pfu in mice, which is much lower than that of other highly pathogenic avian influenza viruses, including H5N1 and H7N9, as reported by others. The reported MLD_50_ values of A/Vietnam/1203/04 (H5N1) and A/Anhui/1/13 (H7N9) are 32 pfu (10^1.5^ pfu) and 2512 pfu (10^3.4^ pfu), respectively^14,21^. However, it is important to note that it is difficult to compare the experimental results based on published papers, as the experimental conditions and procedures can vary among different laboratories. We were not able to compare H5N6/GZ14 with H7N9 or H5N1 virus in parallel due to the limitations in our Biosafety level 3 animal facility. Nevertheless, the dramatically lower MLD_50_ of H5N6/GZ14 suggested the extremely high virulence of this patient-derived H5N6 virus in BALB/c mice. The results in a mouse model concurred with the clinical observations in H5N6-infected patients, which also showed rapid disease progression with ARDS^11,12^. The high fatality rate of H5N6 influenza infection increases the need to better understand the disease and to evaluate better therapeutics.

The occurrence of a “cytokine storm” or the upregulation of pro-inflammatory cytokines and chemokines has been associated with aggravated lung injury and pneumonia in H5N1- and H7N9-infected patients^22–25^. Exacerbated pro-inflammatory cytokine responses were also detected during severe H5N1 and H7N9 virus infections in mice, which contributed to tissue pathology and fatal outcomes^26–28^. In this study, we found that the levels of pro-inflammatory cytokines and chemokines, including MCP-1, MIP-1β, IL-1β, and GM-CSF, increased rapidly at 1 dpi, peaked at 3 dpi, and remained elevated at 5 dpi in the lungs of H5N6/GZ14-infected mice. In contrast, the level of IL-10 significantly decreased at 3 and 5 dpi. IL-10 is known to be an important anti-inflammatory cytokine and serves as a negative regulator of both innate and adaptive immune responses^29,30^. Thus, these findings suggest that dysregulation of cytokines and chemokines is associated with lung injury and high fatality in H5N6-infected hosts.

Although anti-influenza neuraminidase (NA) inhibitors, such as oseltamivir (Tamiflu), have been used for treating influenza viral infection, the drug is only effective in the early stage of infection^31^. The high fatality and rapid disease progression of H5N6 infection in humans indicate a need to improve the treatment regimen. In clinical practice, passive immunotherapy with convalescent plasma from influenza infection survivors has been proven as a viable option for treating H5N1 infection even at a later stage of infection and for treating H1N1 infection^9,10^. However, there are very few H5N6 survivors, and they are unlikely to be suitable for the donation of plasma due to age and health conditions. In the absence of adequate human donors of convalescent plasma, immunoglobulins or polyclonal antibodies from immunized animal sera have been considered^32,33^. It is reported that prepared F(ab′)_2_ fragments from inactivated H5N1-vaccinated horses could provide protection against H5N1 infection in mice^34^. However, there are concerns of severe allergic responses, such as allergic shock, that result from infusion of foreign antigenic proteins. We proposed a concept of immunizing nonhuman primates with the desired antigens to generate antigen-specific IgG, which consists of specific polyclonal antibodies, for use in urgent situations to treat infections with virulent pathogens, such as highly pathogenic AIV infections. Rhesus macaques are genetically more closely related to humans than most other animals. The immunoglobulin genes of rhesus macaques share up to 95% identity with humans^35^. Therefore, rhesus macaques could be a source to provide more human biocompatible IgG for human use in urgent situations. Another solution would be to clone H5N6 neutralizing antibodies or, ideally, broadly neutralizing antibodies to influenza viruses from infected patients and develop monoclonal antibodies as antibody drugs. However, the long timeline and cost for development may make it unfeasible if the number of patients remains small and there is a lack of incentive in the industry.

In this study, we generated a primate-derived, trivalent anti-influenza IgG containing polyclonal antibodies with neutralizing activities against three avian influenza A viruses: H5N1, H7N9, and H5N6. Although the human isolate H5N6/GZ14 is highly virulent in BALB/c mice, the anti-influenza IgG could rescue the infected mice from lethal infection, demonstrating the effectiveness of polyclonal antibodies for treating H5N6 infection. Although we proposed the exploration of immunized macaque IgG and demonstrated its efficacy in treating H5N6/GZ14 lethal infection in mice, more studies need to be done to assess the practicality of this strategy. The availability of pathogen-free rhesus macaques for immunization and methods for disinfecting possible pathogens in purified IgG should improve the safety and usefulness in the future.

In summary, we found that a patient-derived H5N6 isolate is highly pathogenic in BALB/c mice but can be treated with anti-influenza polyclonal antibodies. Our results provide insights into understanding the pathogenicity and treatment of H5N6 infection.

## Materials and methods

### Ethics statement

All murine experiments were performed in an animal biosafety level 3 facility using HEPA-filtered isolators and under the guidance of the Association for Assessment and Accreditation of Laboratory Animal Care International. The experimental protocol was approved by the Committee on the Ethics of Animal Experiments of Animal Biosafety Level 3 (BSL-3) of Guangdong Inspection and Quarantine Technology Center, Guangdong Entry-Exit Inspection and Quarantine Bureau (SYXK20140086).

The rhesus macaques’ study protocol was approved by the GIBH Institutional Animal Care and Use Committee (2013007). The rhesus macaques were housed and handled in accordance with the guidelines set by the Association for the Assessment and Accreditation of Laboratory Animal Care. All steps taken to ameliorate the welfare and to avoid the suffering of the rhesus monkeys were in accordance with the recommendations of the “Weatherall report for the use of nonhuman primates”.

### Viruses

A/Guangzhou/39715/2014 (H5N6) (H5N6/GZ14) (GenBank No. KP765785~KP765792) was initially isolated from a throat swab specimen collected from an adult male patient with known poultry exposure^8^. The virus isolate was obtained by inoculating the allantoic sac and amniotic cavity of approximately 10-day-old specific pathogen-free (SPF) embryonated chicken eggs. All operations involving live H5N6 virus were carried out in a BSL-3 facility at the Guangdong Inspection and Quarantine Technology Center.

The recombinant influenza virus rH5N6/GZ14 was generated by eight-plasmid-based reverse genetics in the background of A/Puerto Rico/1934 (PR8) containing HA and NA sequences of H5N6/GZ14. The other two “6 + 2” recombinant viruses, A/Vietnam /1194/2004 (H5N1) (rH5N1/VN04) and A/Anhui/1/2013 (H7N9) (rH7N9/AH13), were generated by the same method in our previous studies^36,37^. These recombinant viruses were propagated in embryonated chicken eggs and then purified by using a sucrose gradient centrifugation as described previously^36^.

### Immunization of rhesus macaques with inactivated recombinant influenza viruses

Purified rH5N1/VN04, rH7N9/AH13, and rH5N6/GZ14 were inactivated by treatment with 0.1% formalin at 4 °C for 3 days to prepare the inactivated vaccine antigen. The HA protein content of inactivated vaccine was estimated to make up ~30% of the total protein of inactivated vaccine, which was measured by BCA protein assay kit (Pierce). Six healthy male Chinese rhesus macaques, previously immunized with a H7N9 vaccine^36^, were immunized with a mixture of inactivated recombinant viruses rH5N1/VN04, rH7N9/AH13, and rH5N6/GZ14, containing 15 μg HA of each with aluminum hydroxide via intramuscular injection. Four weeks later, these macaques were immunized again with the same inactivated recombinant viruses. Considering the welfare of animals, approximately 5 ml of blood was collected from each macaque at 2, 6, 10, 14, and 18 weeks after the last immunization. The sera collected at different time points from these macaques were frozen at −80 °C. After the samples from all the time points were collected, the serum samples were then pooled together for purification of IgG. The HI and neutralization titers were determined.

### Purification of anti-influenza IgG

A total volume of 50 ml of hyperimmune sera pooled from immunized rhesus macaques was purified by Protein A-Sepharose affinity chromatography with ÄKTApurifier 10 according to the manufacturer’s instructions (GE Healthcare). Briefly, the hyperimmune sera were centrifuged at 12 000 × *g* for 15 min to remove particulate matter. The supernatant was diluted with 10 column volumes of physiological saline and then filtered through 0.45 μm filter before loading on the Vantage column (Millipore), which was packed with rProtein A-Sepharose Fast Flow, at a volumetric flow rate of 3.5 ml/min. The loaded serum sample was washed with physiological saline until the ultraviolet absorbance reached at 20 mAU and then eluted with 0.1 M sodium citrate (pH 3.5). The collection samples were neutralized with 1 M Tri-HCl (pH 9.0) to ensure the eluted immunoglobulin was approximately neutral. Total protein content of purified IgG was determined by a BCA protein assay kit (Pierce) according to the manufacturer’s instructions. Samples before and after IgG purification were analyzed using Coomassie blue stained sodium dodecylsulfate polyacrylamide gel electrophoresis (SDS-PAGE) gels. The HI and neutralization titers of purified IgG were determined.

### SDS-PAGE analysis

Standard one-dimensional SDS-PAGE was carried out as described by Laemmli^38^. A total volume of 10 μl of diluted samples, with 2.5 μl of loading dye with 2% (vol/vol) β-mercaptoethanol as reducing agent, was heated to 95 °C for 5 min and then loaded onto a 10% acrylamide gel. The gel was run at 20 mA for approximately 90 min until the tracking dye just ran out of the gel, followed by Coomassie Blue staining.

### HI assay

Standard HI assays were performed on post-immune rhesus macaque sera and purified IgG using 1% chicken erythrocytes against recombinant rH5N1/VN04, rH7N9/AH13, and rH5N6/GZ14 according to standard protocols^39^.

### Microneutralization assay

The neutralizing antibody titer of purified immunoglobulins was determined by microneutralization (MN) assays as previously described with minor modifications^40^. In brief, 50 μl of influenza virus containing 100 TCID_50_ was incubated with 50 μl of twofold dilutions of the cholera filtrate-treated serum for 1 h at room temperature. After incubation, the virus-serum mixtures were transferred to 96-well plate containing an Madin-Darby Canine Kidney cell (MDCK) monolayer and then incubated for 2 h at 37 °C and 5% CO_2_. The virus-serum mixtures were removed from the wells and then washed once with phosphate buffered saline (PBS). The cells were incubated at 37 °C for 2 days in Dulbecco’s modified Eagle’s medium (DMEM) supplemented with 0.3% bovine serum albumin (BSA) and 1 μg/ml of TPCK-treated trypsin. The plates were fixed with 4% paraformaldehyde for 10 min. The presence of viral protein was detected by ELISA with a monoclonal antibody (Abcam) to the influenza A nucleoprotein. The MN titer was defined as the highest dilution of serum that resulted in 50% neutralization of 100 TCID_50_ of virus in MDCK cell cultures.

### Establishment of the H5N6/GZ14 mouse model

SPF female BALB/c mice (Vital River Laboratory, Beijing) were used. Eight-week-old female BALB/c mice with six mice for each group were infected intranasally with 1, 10,100, and 1000 pfu of H5N6/GZ14 in 50 μl of PBS. Mice were monitored for signs of illness, weight loss, and mortality for up to 14 days. Mice were euthanized when the loss of original body weight exceeds 25%. To avoid excess disturbance, the mice were weighted every other day. The dose required to kill 50% of the mice (MLD_50_) was calculated by the Reed-Muench Method^41^.

To determine the extent of viral replication in mice, mice were infected with 10 MLD_50_ of H5N6/GZ14. The mice (*n* = 3) were sacrificed at 1, 3 and 5 dpi for virological assays. Blood samples were collected through intracardiac puncture after anesthetization, and then, mice were euthanized for dissection. The blood was allowed to clot, and the serum was separated by centrifugation and stored at −80 °C. The lung, heart, liver, spleen, kidney, and brain were collected. The organs were weighted and homogenized in 1 ml cold PBS, and virus replication titers were determined by plaque formation assays. The limit detection for all tissues was 10 pfu/ml. Parts of lungs of infected mice were fixed for the histopathological analysis.

### Plaque formation assay

Confluent MDCK cell monolayers in six-well plates were infected with 10-fold dilutions of virus-containing samples (allantoic fluid or tissue homogenate) in a total volume of 1.0 ml DMEM containing 0.3% BSA for 1 h at 37 °C. The wells were aspirated and washed once with PBS to remove residual virus. Each well was then immediately covered with an agar overlay (final concentration: 1% agar; 1× modified Eagle’s medium; 0.3% BSA; and 1 µg/ml TPCK trypsin). The plates were then incubated at 37 °C with 5% CO_2_ for 3 days. The overlays were carefully removed, and the cells were stained with crystal violet. The number of pfu was counted and recorded.

### Measurement of influenza viral RNA in the serum sample

Total RNA from 100 μl of serum sample was extracted using a QIAamp viral RNA mini kit (Qiagen). Complementary DNA was prepared using a Primescript cDNA synthesis kit (TaKaRa) and uni-12 primer (5′-AGCA/GAAAGCAGG-3′), which is based on the consensus 3′-sequence of the vRNA end sequences of influenza A virus. The relative amounts of the H5N6 virus genome were quantified using absolute real-time qPCR assays with a fixed quantity of 20 ng cDNA per reaction. Plasmid standards were developed using a pMD-18 T vector (TaKaRa) containing a HA fragment of H5N6/GZ14. Plasmid copy numbers were determined by calculating the molecular weight of the cloned plasmid. For absolute quantitation of viral copies, standard curves were constructed using an amplification of a 180-base pair fragment of the HA gene and 10-fold serial dilutions of the plasmids.

The qPCR was carried out in a 25 µl ready-to-use SYBR Green reaction mixture (TaKaRa) with gene-specific primers for HA (5′-ATCAGAGTGCCGGAATGGTC-3′ and 5′- ATGGACATGCTGCGCTCAC-3′) in a CFX96 Touch Real-Time PCR Detector (Bio-Rad). The PCR conditions were 95 °C for 30 s and 40 cycles of 95 °C for 5 s, 55 °C for 30 s, and 72 °C for 10 s. Duplicate reactions were run for each sample. The results of qPCR assays were analyzed using CFX manager software version 3.1 supplied with the CFX96 Touch (Bio-Rad).

### Histopathological analysis of lung tissues

Lung tissues from infected and mock mice at indicated time points were fixed in 10% neutral buffered formalin and embedded in paraffin. Tissue sections (5 μm) were stained with hematoxylin and eosin and analyzed under light microscopy.

### Detection of cytokines and chemokines

Expression levels of IFN-α, TNF-α, MIP-1α, MIP-1β, MCP-1, IFN-γ, IL-6, and IP-10 in the lung homogenates from 10 MLD_50_ of H5N6/GZ14-infected mice on 1, 3 and 5 dpi were quantitatively determined by use of ELISA kits (eBioscience) according to the manufacturer’s protocol (assay sensitivity, 15.6 pg/ml).

### Treatment of H5N6/GZ14-infected mice with anti-AIV IgG

Eight-week-old female BALB/c mice, six mice/group, were infected intranasally with 2 MLD_50_ of H5N6/GZ14 virus under anesthesia with isoflurane. Twenty-four hours later, mice were intraperitoneally injected with either 1 or and 3 mg/mouse of anti-AIV IgG. The control group received purified IgG from macaques immunized with an uncorrelated antigen. The mice were observed for up to 14 days and weighted every other day.

### Statistical analysis

Statistical significance was determined using unpaired, two-tailed Student’s *t*-tests with the GraphPad Prism 5 software package (GraphPad Software). *P* values < 0.05 were considered significant.
